# Heteronuclear Micro-Helmholtz Coil Facilitates µm-Range Spatial and Sub-Hz Spectral Resolution NMR of nL-Volume Samples on Customisable Microfluidic Chips

**DOI:** 10.1371/journal.pone.0146384

**Published:** 2016-01-05

**Authors:** Nils Spengler, Jens Höfflin, Ali Moazenzadeh, Dario Mager, Neil MacKinnon, Vlad Badilita, Ulrike Wallrabe, Jan G. Korvink

**Affiliations:** 1 Institute of Microstructure Technology (IMT), Karlsruhe Institute of Technology (KIT), Eggenstein-Leopoldshafen, Germany; 2 Freiburg Institute for Advanced Studies (FRIAS), University of Freiburg, Freiburg, Germany; 3 Laboratory of Simulation, IMTEK–Department of Microsystems Engineering, University of Freiburg, Freiburg, Germany; 4 Laboratory for Microactuators, IMTEK–Department of Microsystems Engineering, University of Freiburg, Freiburg, Germany; The Francis Crick Institute, UNITED KINGDOM

## Abstract

We present a completely revised generation of a modular micro-NMR detector, featuring an active sample volume of ∼ 100 nL, and an improvement of 87% in probe efficiency. The detector is capable of rapidly screening different samples using exchangeable, application-specific, MEMS-fabricated, microfluidic sample containers. In contrast to our previous design, the sample holder chips can be simply sealed with adhesive tape, with excellent adhesion due to the smooth surfaces surrounding the fluidic ports, and so withstand pressures of ∼2.5 bar, while simultaneously enabling high spectral resolution up to 0.62 Hz for H_2_O, due to its optimised geometry. We have additionally reworked the coil design and fabrication processes, replacing liquid photoresists by dry film stock, whose final thickness does not depend on accurate volume dispensing or precise levelling during curing. We further introduced mechanical alignment structures to avoid time-intensive optical alignment of the chip stacks during assembly, while we exchanged the laser-cut, PMMA spacers by diced glass spacers, which are not susceptible to melting during cutting. Doing so led to an overall simplification of the entire fabrication chain, while simultaneously increasing the yield, due to an improved uniformity of thickness of the individual layers, and in addition, due to more accurate vertical positioning of the wirebonded coils, now delimited by a post base plateau. We demonstrate the capability of the design by acquiring a ^1^H spectrum of ∼ 11 nmol sucrose dissolved in D_2_O, where we achieved a linewidth of 1.25 Hz for the TSP reference peak. Chemical shift imaging experiments were further recorded from voxel volumes of only ∼ 1.5nL, which corresponded to amounts of just 1.5 nmol per voxel for a 1 M concentration. To extend the micro-detector to other nuclei of interest, we have implemented a trap circuit, enabling heteronuclear spectroscopy, demonstrated by two ^1^H/^13^C 2D HSQC experiments.

## Introduction

Nuclear magnetic resonance (NMR) is, despite its great utility as a non-invasive and non-destructive instrument, one of the least sensitive of the analytical techniques. Recent advances in probe design, RF electronics, and most importantly, superconducting magnets delivering large increases in field strengths, have greatly increased the sensitivity and resolution of NMR spectroscopy. However, NMR still falls behind mass spectrometry in molar sensitivity, and is usually reserved for samples that can be obtained in reasonable quantities. Sometimes, the lack of sufficient sample completely prevents NMR investigation, despite the advantages it would provide to the analyst, *e.g*., straightforward quantification, or insights on the molecular structure of a substance. For such cases, the ability to perform NMR spectroscopy on very small sample volumes (microliters and below, and at native concentrations) within a reasonable time would be a complete game changer, and could lead to a significant move forward in analytical research.

For mass or volume-limited samples (μg or μL and below) it has been shown [1] that NMR signal acquisition can be significantly accelerated when using micro-NMR coils (diameters ≲1 mm) due to their increased sensitivity. However, miniaturised systems still lack a number of features that macroscopic systems offer, which inhibit the wide-spread use of small-scale detectors:

- Apart from a few exceptions [2], non-commercial [3] and commercial micro-NMR probes such as CapNMR [4] (Protasis MRM, Savoy, IL, USA) are monolithic constructions. In contrast, macroscopic probes consist of a separate coil and a sample container (tube), which is more practical. Current micro detectors need to be cleaned prior to reuse, which can be problematic.
- Many publications on MEMS-fabricated micro-coils for magnetic resonance [5] concentrate on detector designs while neglecting sample handling or interfacing of the sample chamber. As a consequence, sample storage or recovery is difficult.
- In several popular designs [6, 7], optical inspection of the sample inside the probe is difficult, as the view is blocked by opaque materials. It is therefore difficult to check whether a probe is only partially filled, or whether an air bubble is trapped in the sensitive coil volume, issues which will cause susceptibility broadening at the liquid-gas interface.
- The most prominent micro-coil geometry, a solenoid, has to be operated perpendicular to the *B*_0_-field, and requires immersing the probe in a susceptibility-matched liquid [6] in order to achieve sub- Hz resolution, whereas the saddle construction of macroscopic NMR coils reduces *B*_0_-field distortions *per se*, and hence results in narrower spectral peaks without additional effort.

The low sensitivity of NMR spectroscopy becomes even more pronounced for nuclei with a low natural abundance such as ^13^ *C* (1.1%) as well as with a low gyromagnetic ratio *γ*, which leads to a smaller magnetic moment, a weaker induced signal, and often to an increased *T*_1_ relaxation time which increases the minimum repetition time required between two NMR acquisitions.

Polarisation transfer techniques are one possible way to compensate for the reduced sensitivity, where the insensitive spin population is detected indirectly or directly, by means of polarisation transfer from a second, more sensitive spin (usually the hydrogen proton). In case of *J*-coupling, it requires addressing both species simultaneously using a double-resonant probe. Heteronuclear two-dimensional (2D) NMR experiments, such as “insensitive nuclei enhanced by polarisation transfer” (INEPT) or “heteronuclear single quantum coherence” (HSQC), employ polarisation transfer via scalar *J*-coupling. For example, one can transfer spin order from ^1^H to ^13^C and increase sensitivity of the ^13^C spins by a factor of 4 for INEPT and 32 for HSQC [8]. Additionally, 2D techniques are inevitable when identifying cross-peaks for the purpose of structure elucidation.

To compensate for the drawbacks listed above, we have previously reported on a modular concept, which incorporated a micro Helmholtz coil pair and exchangeable, microfluidic sample inserts [9]. However, the probe could only perform homonuclear experiments, and suffered from several deficits, such as a low fabrication yield due to large variations in layer thicknesses of the dispensed photoresist, while tight sealing or active perfusion of the sample containers was difficult due to the microfluidic ports being located at the edges of the chips. Spectral peaks showed distortions due to the size and geometry of the sample chamber. The present paper reports on the optimisation of the fabrication process of the micro Helmholtz coil pair chip to simplify manufacturing while simultaneously increasing the yield and passive shimming. We have further reworked the layout and the fabrication of the sample inserts to facilitate application-specific designs with additional microfluidic or electromagnetic functionality, enable straightforward sealing of sample chambers, and significantly improved spectroscopic performance. The probe was also rendered double-resonant to enable heteronuclear 2D experiments.

While geometric decoupling, *i.e*., the arrangement of two detector coil pairs at right angles to each other, is only straightforward if one [10] or both coils [11] are of macroscopic size, but is difficult at small scales due to geometrical and fabrication constraints, we have implemented a trap circuit [12–14] for double-tuning the probe to the proton and carbon Larmor frequencies.

## Materials and Methods

### Design

**Fig 1:**
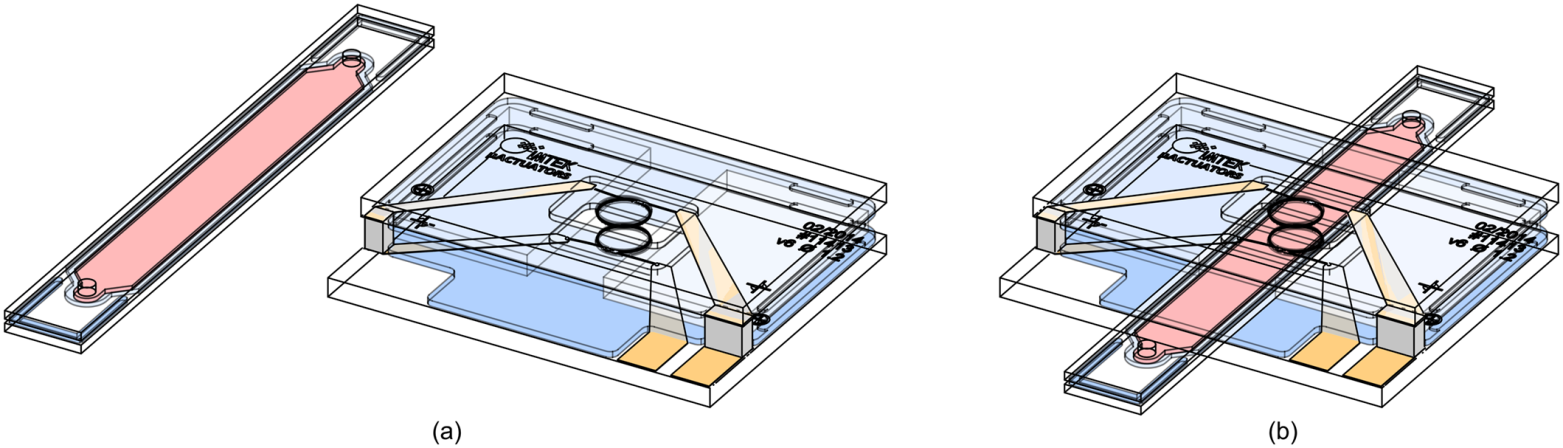
Technical drawing of the setup. It consists of a (10 × 8 × 1.88)mm^3^ micro-NMR probe, and of a (2 × 16 × 0.6) mm^3^ exchangeable microfluidic application-specific sample insert (ASSAI) with a total sample volume of 2 μL, with the analyte indicated in red.

A 1.2 mm diameter micro-fabricated Helmholtz coil pair was employed for all the experiments performed, and was used in combination with custom made, glass-based, application-specific sample inserts (ASSAIs) that can be exchanged, as shown in Fig 1. The coil pair was integrated into a (10 × 8 × 1.88)mm^3^ chip stack (*w* × *l* × *h*) and contains a (2 × 0.55)mm^2^ slot opening (*w* × *h*) to accommodate the ASSAI.

In our first prototype [9], the spacing between the coil pair was determined by a defined volume of liquid photoresist, which was prone to thickness variations as it was dependent on the exact amount of resist to be dispensed, as well as on accurate levelling during curing to prevent re-flow. A smooth surface was only achieved, when the coils were covered by at least 100 μm of resist. Keeping the Helmholtz coil pair condition in mind (distance equals radii), this fact limited the minimum coil diameter to ∼ 1.5 mm and restricted the maximum number of turns per coil due to the resulting self-resonance frequency, which decreases for increasing number of turns and coil diameters. We have therefore replaced the liquid resist by dry film resist (DFR), which does not rely on levelling during curing while having typical thickness variations < 5 μm [15]. The toroidal-shaped opening around the post enables wirebonding of the coils and subsequent encapsulation for fixation and protection, while the edges of the surrounding DFR act as pinning barriers for the liquid encapsulation material and hence enable smooth surfaces without additional material on top of the coils, which allowed the reduction of the coil diameter and the increase of the number of windings.

To circumvent further thickness variations, which may occur by melting and re-solidification of the laser-cut PMMA spacer in case of improper laser parameters, we have additionally substituted the lasered polymer spacers by spacers that were diced out of a glass wafer. The latter was temporary glued on a sacrificial silicon wafer prior to dicing, to allow for deep cuts and burr-free edges.

The round, microfluidic sample chamber was replaced by an elongated, rectangular-shaped chamber geometry that is oriented along the *B*_0_-field, similar to NMR tubes, while the horizontally running microfluidic leads were omitted. We further introduced laser-drilled, microfluidic ports at the top surface to enable reliable sealing simply using Scotch tape, which was difficult for the former design due to the side entrances and non-flush surfaces caused by dicing burrs nearby. In contrast, the improved design enables gas-tight sealing using standard O-rings and therefore straightforward external interfacing, which was not possible before.

To reduce distortions of the *B*_0_-field and hence to improve spectral resolution, the slot opening was rotated by 90° for aligning the rectangular-shaped ASSAI in parallel to the orientation of the *B*_0_-field. Steps in magnetic susceptibility, *e.g*., near the microfludic ports, were therefore relocated to cross the *B*_0_-field further away from the sensitive volume of the micro-coil.

### Fabrication

#### ASSAI

**Fig 2:**
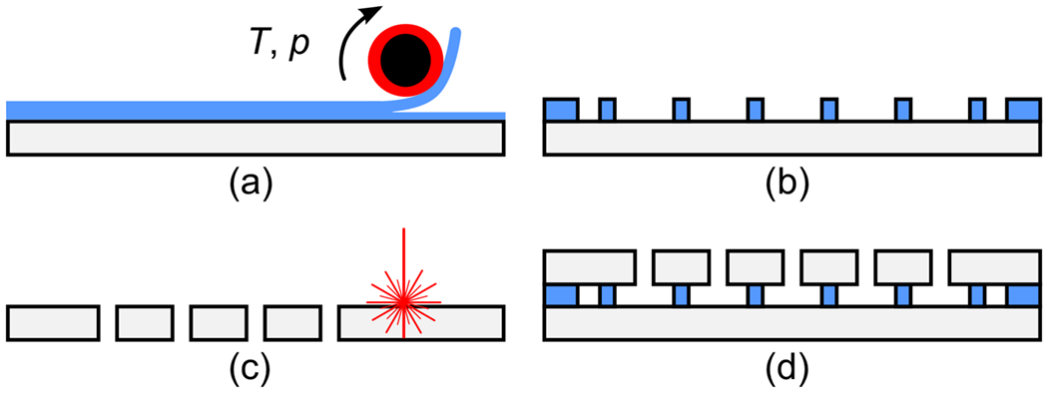
Schematic illustration of the ASSAI fabrication process. (a) Multiple lamination of DFR on bottom wafer. (b) UV exposure of dry film resist and development. (c) Laser-drilling of top wafer. (d) Full-wafer bonding.

ASSAIs were manufactured from two (208 ± 20) μm thick, D 263 T (Schott AG, Mainz, Germany) 4-inch diameter float glass wafers, as indicated in Fig 2 and took around 3h—4h in total.

At first, microfluidic channels with a total nominal height of 110 μm were patterned on the bottom substrate in two layers of Ordyl SY355 DFR (Elga Europe s.r.l., Milano, Italy) using a MyJOY 12 hot roll laminator (GMP Prographics GmbH, Polch, Germany), as illustrated in Fig 2a and 2b. To improve the adhesion of the first layer, the surface of the wafer was first activated in an *O*_2_-plasma (Diener electronic GmbH + Co. KG, Calw, Germany) at 0.3 mbar, 40°C, and 20 W at 40 kHz. Lamination was done at a speed of 1 cms^−1^, a pressure of 1 bar, and a temperature of approximately 100°C. A low exposure dose of 180 mjcm^−2^ was applied using a MA6 mask aligner (Karl Süss MicroTec AG, Garching, Germany) for minimum cross-linking while a hard baking step was omitted to enable subsequent wafer bonding.

Microfluidic ports with a diameter of 400 μm were pre-drilled into the top wafer using a TruMark 6330 UV-laser (Trumpf, Ditzingen, Germany) as indicated in Fig 2c. Prior to laser drilling, a highly absorptive, sacrificial polymer layer such as Scotch or dicing tape had to be applied onto the substrate as an absorbing layer to initiate laser cutting of the glass. Each wafer contained 129 chips in total, resulting in 258 drilled holes.

**Fig 3:**
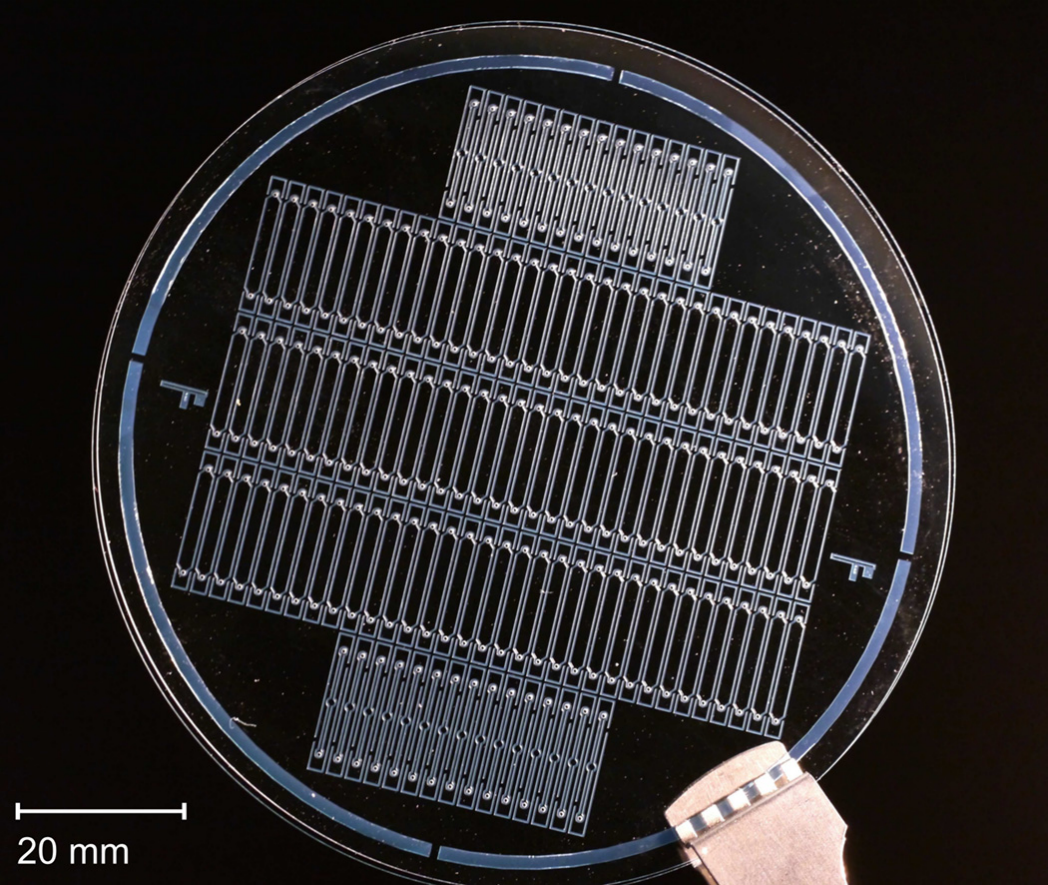
Photograph of bonded wafers before dicing. The stack contains 129 ASSAIs.

Both substrates were bonded in a full wafer bonding process according to published methods [16, 17] using a SB6 substrate bonder (Karl Süss MicroTec AG, Garching, Germany), depicted in Fig 2d. Upon plasma activation of the top wafer containing the drilled holes, substrates were aligned manually and fixed using the clamps of the SB6 chuck. In case higher alignment precision is required (accuracy < 100 μm), optical alignment can be done using the mask aligner as well. The clamped wafers were loaded into the machine and a tool pressure of 2.4 bar was applied for 30 min at 95°C. Before dicing, the stack was hard-baked for 2 H at 150°C. A photograph of bonded wafers with a yield of 100% containing 129 inserts, (2 × 16 × 0.5) mm^3^ (width × length × height) in size, is shown in Fig 3.

#### Probe

**Fig 4:**
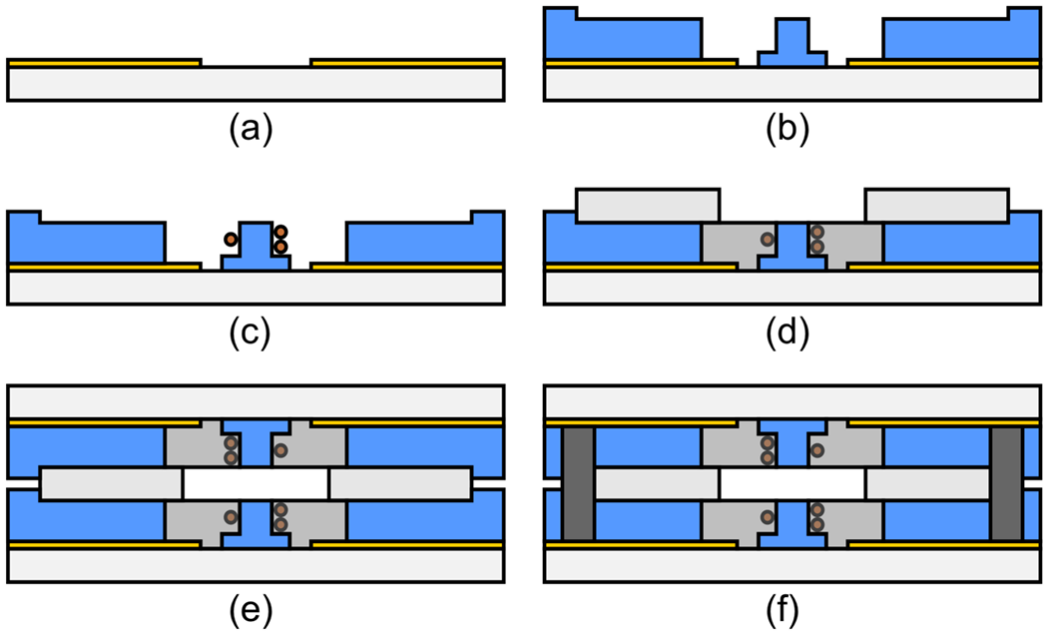
Schematic illustration of the probe fabrication process. (a) Electroplating of Au. (b) Patterning of dry film resist. (c) Wirebonding of micro-coils. (d) Encapsulation of coils and glueing of glass spacers. (e) Flip-chip bonding of top and bottom chips. (f) Soldering of electrical interconnects.

The fabrication of the probe is sketched in Fig 4 and was performed approximately within 1.5 working days. The detailed steps are outlined as follows.

Micro-machining starts with a 0.5 mm thick, 4-inch diameter Borofloat 33 borosilicate glass wafer (Schott AG, Mainz, Germany). A transparent glass substrate was chosen to enable optical inspection of the layer stack and the enclosed sample during use. In addition, using a non-conductive substrate prevents the formation of additional conductive paths and the induction of eddy currents in the vicinity of the coil, both of which degrade the electrical performance of the sensor [18]. Moreover, the low coefficient of thermal expansion (CTE) of 3.25 ppmK^−1^ is advantageous because of thermal extrema occurring during fabrication, *e.g*., during wirebonding, which is performed at substrate temperatures of 150°C.

A 15/150nm Cr/Au seed layer was first evaporated on the substrate. Subsequently, hexamethyldisilazane (HMDS) was applied before 20 μm AZ9260 positive-tone photoresist (MicroChemicals GmbH, Ulm, Germany) was patterned according to the manufacturer’s datasheet as a mould structure for electro-deposition to form conductive tracks and pads for wirebonding. 10 μm Au was electroplated (Fig 4a) in order to fully exploit the skin depth *δ*_Au_(500 MHz) = 3.52 μm and hence to minimise noise caused by resistive losses. After stripping the mould and wet-etching the Au seed layer, 2 μm ma-N 1420 negative tone photoresist (Micro Resist Technology GmbH, Berlin, Germany) was spun and patterned according to the manufacturer’s recipe to form markings and alignment structures in the Cr-layer, which was subsequently wet-etched. Finally, the ma-N 1420 layer was stripped in acetone and the substrate was cleaned in isopropyl alcohol (IPA) and deionized water before spin-drying. All etching and stripping steps were performed using megasonic agitation by placing a petri dish containing the required chemical on a megasonic transducer, immersed in H_2_O, to ensure uniform wetting of the surface and homogeneous etching rates.

Afterwards, 55 μm thick Ordyl SY355 DFR was laminated onto the substrate using the process parameters given in the previous section. In total, four sheets of resist were alternately laminated and exposed, resulting in a total nominal resist thickness of 220 μm, as shown in Fig 4b. After each lamination step, a 1 min post lamination bake was performed at 85°C on a hotplate. During the post lamination bake, the resist completely flattens and small wrinkles vanish, which may have occurred during lamination.

In the first Ordyl SY355 layer, a cylindrical post base plateau was structured with an outer diameter (OD) of 1.275 mm using foil masks (Koenen GmbH, Ottobrunn, Germany) at an exposure dose of 180 mjcm^−2^, which was needed to suspend the micro-coils later on and to fix their vertical positions. Two more layers of Ordyl SY355 were laminated and posts with 1.175 mm OD were patterned at 225 mjcm^−2^, resulting in a nominal height of 165 μm. Alignment bars were patterned into the final layer at 225 mjcm^−2^ to provide mechanical alignment for the glass spacers glued after wirebonding.

In the next step, micrcocoils with two windings were wirebonded [9] from insulated 25 μm diameter copper wire, at 150°C using a modified industrial ball-wedge wirebonder *ESEC WB 3100* (Oerlikon AG, Zurich, Switzerland), as shown in Fig 4c. An approximate clearance of one wire diameter remained between the uppermost piece of the wire and the edge of the post. An SEM closeup of a wirebonded coil supported on the post base plateau is shown in S2 Fig.

The coils were encapsulated in a rigid, transparent polyurethane (WC-783, BJB Enterprises Inc., Tustin, CA, USA), which has been characterised for its MR suitability [19]. For the encapsulation process, which was performed under a microscope equipped with a ring-light illumination, a pressure-driven, semi-automatic dispensing machine was used (DX-250, Metcal, Hampshire, UK) in combination with a size 32 dispensing needle.

Spacers were fabricated from 4-inch, 550 μm thick float glass wafers (Anti-Newton, AGC Glass Europe, Louvain-la-Neuve, Belgium), diced into (3.7 × 5.6) mm^2^ pieces (width × length), to form a slit with a width of 2 mm to accommodate the micro-fabricated ASSAIs as introduced in Fig 1. In contrast to laser-cutting of polymers, a rigid, brittle, high-melting material such as glass ensures planar surfaces without melting or bending during machining, and therefore retains tolerances. To accommodate the nominal height of the ASSAI of 526 μm, the slot height defined by the spacers was chosen to be slightly larger than the thickness of the insert.

The stack was assembled in a two-step process using a stamp-and-stick gluing technique [20]. At first, 2mL WC-783 polyurethane was dispensed in a polystyrene petri dish, clamped on a spin-coater using vacuum and spun 15 s at 500 revolutions per minute (RPM) and 60 s at 2000 RPM to achieve a uniform layer of ∼ 5 μm thick polyurethane. The glass spacers were then first placed inside the petri dish and then transferred onto the bottom chips and gently pushed to the corners towards the alignment bars using plastic tweezers (Fig 4d).

The bottom chips were cured at 50°C for 6 H before the procedure was repeated. Here, the bottom chips with the already fixed glass spacers were placed upside down into the petri dish, *i.e*., with the glass spacers touching the liquid polyurethane layer, and afterwards flipped and placed under a microscope. Subsequently, top chips were placed upside down on top of the glass spacers and gently pushed from the back towards the front of the chips until the upper alignment bars again touched the glass spacers (Fig 4e). The stack was again cured at 50°C for 6 H to cross-link the polyurethane. After the glueing step, electrical connections between the bottom and the top chip were established by soldering (Fig 4f). A polished cut of an assembled chip stack is shown in S3 Fig.

### Electrical setup

The probe was characterised with a 1-port measurement using an E4991A RF impedance analyser (Agilent, Santa Clara, CA, USA), connected to a Summit 9000 RF probe station (Cascade Microtech GmbH, Thiendorf, Germany). Prior to the actual measurement, the setup was calibrated in an open/short/load calibration measurement over the entire frequency range using an impedance standard substrate to cancel deviations and offsets induced by the equipment, *e.g*., connectors, cables and the ACP40-A-SG-500 probe tip (Cascade Microtech GmbH). At 500MHz, the proton Larmor frequency of our 11.7 T NMR scanner, we measured a probe inductance *L*_*p*_ = 27 nH and a direct current (DC) serial resistance *R*_p_ = 0.8Ω.

The chip was glued on a 0.5 mm thick printed circuit board (PCB) using Araldite 2020 epoxy (Huntsman Advanced Materials GmbH, Basel, Switzerland) based on the stamp-and-stick gluing technique described above. For the first homonuclear experiments, the probe was connected to a balanced tuning and matching circuit, tuned to 500MHz and matched to 50Ω, where the parallel tuning capacitor *C*_*t*_ was 0.5 pF and the two matching capacitors *C*_*m*1_ and *C*_*m*2_ were 2.7 pF and 6.8 pF, respectively, each connected in series at either ends of *C*_*t*_. We used non-magnetic, high-*Q*_*c*_ SMD capacitors (SRT Micro Céramique, Vendôme, France), in order to minimise *B*_0_-field distortions from the circuitry.

Finally, the probe was electrically connected to an *LC*-based, triple-resonant RF trap circuit whose design was first proposed by Kan *et al*. [21]. However, due to the two channel limitation of our Micro 5 probe base (Bruker), only two channels were implemented, and adapted to the higher field strength in our case according to the scheme shown in S1 Fig, to realise an X-nucleus channel in addition to the ^1^ H channel. The probe was double-tuned to ^1^H/^13^C, which, at *B*_0_ = 11.7 T, corresponds to Larmor frequencies of *ω*_1h_/2*π* = 500 MHz and *ω*_13c_/2*π* ≈ 125 MHz using the scheme introduced above. For the passive tuning and matching components we used the values listed in Table 1.

**Table 1:** Employed values of the components illustrated in S1 Fig. Capacitances *C*_*i*_ are given in pF, while inductances *L*_*j*_ are given in nH.

Part	*C*_1_	*C*_2_	*C*_3_	*C*_4_
Value	0.4–0.7	0.5–1.1	56	6.8
Part	*C*_5_	*C*_6_	*C*_7_	*L*_4_ = *L*_5_
Value	4.3	1–12	20–32	10

**Fig 5:**
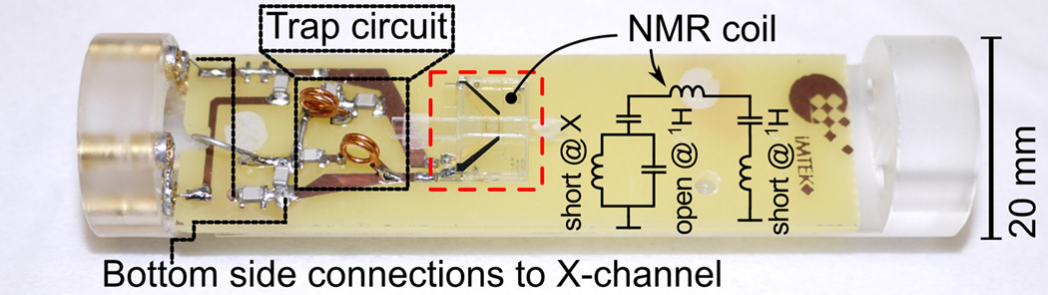
Helmholtz microcoil integrated onto a PCB. The setup was mounted on a custom made sample holder, designed to fit onto a commercial Bruker Micro 5 probe base.

The decoupling efficiency between both ports was measured by determining the reverse voltage gain *S*_12_ using an E5071B network analyser (Agilent). At 125MHz, an *S*_12_ of -40 dB was measured while *S*_12_ was -49 dB at 500MHz. A photograph of the assembled probe, mounted on a custom PMMA holder that fits the pin header of the Micro 5 probe base is illustrated in Fig 5.

### Magnetic Resonance

All magnetic resonance experiments were performed on an Avance III NMR system operating at a ^1^H frequency of 500.13MHz (Bruker, Rheinstetten, Germany). The one-dimensional (1D) spectrum was obtained with a single pulse excitation experiment, collecting 128 averages each with 16384 points over a sweep width of 6 kHz (12 ppm) using a 90° pulse length of 7 μs and a recycle delay of 3 s (experiment time *t*_exp_ = 12 min, 17 s). The resulting FID was Fourier transformed after exponential multiplication equivalent to 0.5 Hz line broadening. The software package Chenomx NMR Suite 8.1 (Chenomx, Edmonton, Canada) was used for further spectral processing (phase and baseline correction, reference deconvolution).

Two 2D HSQC spectra were acquired, the first of pure ethanol (CH_3_CH_2_OH, Sigma-Aldrich) and the second of 1 M sucrose (C_12_H_22_O_11_, Sigma-Aldrich) in D_2_O. For the ethanol experiment, the ^1^H and ^13^C sweep widths were 10 ppm and 118 ppm, respectively. The number of points were 1024 and 256 in the F2 and F1 dimensions, and 64 averages were collected for a total experiment time *t*_exp_ = 5 h, 33 min. For the sucrose experiment, the ^1^H and ^13^C sweep widths were 6.7 ppm and 197 ppm, respectively. The number of points were 1024 and 256 in the F2 and F1 dimensions, and 128 averages were collected for a total experiment time *t*_exp_ = 12 h. Both spectra were post-processed using using the 2D NMR processor ACD/Labs 12.0 (ACD/Labs, Toronto, Canada).

All spectra were referenced to 0 ppm using 0.1 M deuterated trimethylsilyl propanoic acid (TSP) as an internal standard.

## Results

### Probe characterisation

Pulse parameters were determined by performing a nutation experiment of a H_2_O-filled ASSAI using a constant power of 1 W, 5 kHz sweep width, 16384 points and the pulse length *τ* varying from 0 μs–30 μs in steps of 0.5 μs, while a flip angle *α* = *π*/2 (90°) was found at *τ*_*π*/2_ = 7 μs. These values correspond to a *B*_1_-field amplitude

$$B_{1}=\frac{\alpha }{\gamma \tau }$$ (1)

of 0.84 mT and a probe efficiency

$$\eta_{p}=\frac{B_{1}}{i\sqrt{R_{p}}}=\frac{B_{1}}{\sqrt{P_{p}}}$$ (2)

of 0.84 mTW^−1/2^, where *i* is the unit current, *P*_p_ the applied power, and *B*_1_/*i* is the coil sensitivity [22]. As a comparison, for a 10 mm saddle coil (Bruker) *τ*_*π*/2_ was determined to be 90 μs at 1 W, which corresponds to *B*_1,10 mm_ = 0.065 mT and *η*_*p*, 10 mm_ = 0.065 mTW^−1/2^ and consequently shows a sensitivity which is more than one order of magnitude lower, confirming the increased sensitivity of the micro-coil, which scales inversely with the coil diameter [23].

**Fig 6:**
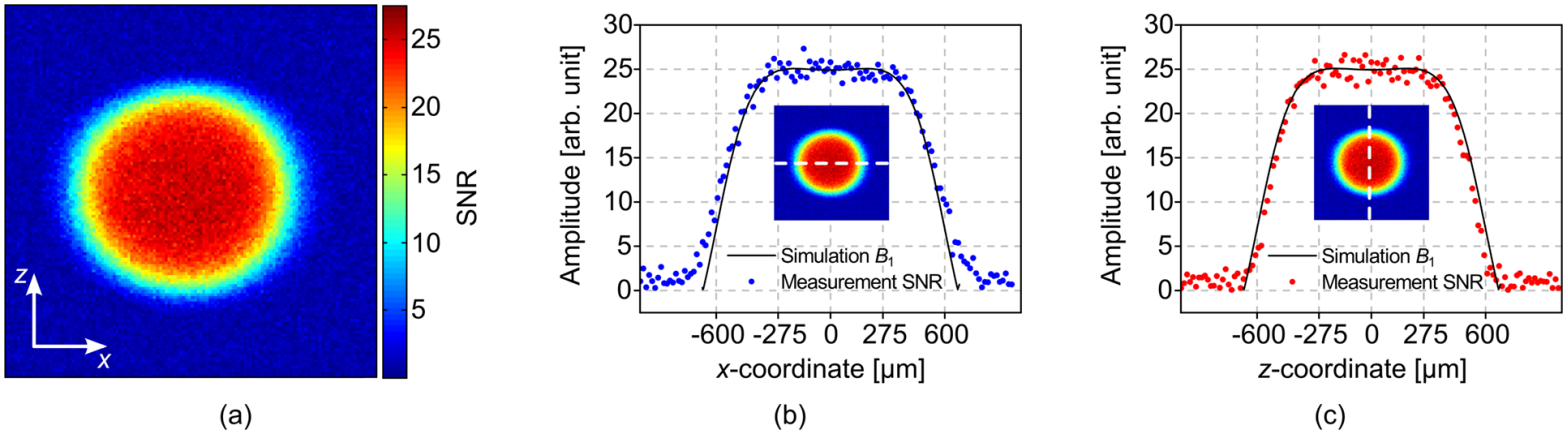
SNR map and extracted profiles along two perpendicular axes. (a) SNR map, coronal *xz*-plane. (b) Extracted SNR values along the *x*-axis and simulated *B*_1_ profile. (c) Extracted SNR values along the *z*-axis and simulated *B*_1_-field profile.

Due to the proportionality of the signal-to-noise ratio SNR ∝ *B*_1_ [23], the spatial distribution of the *B*_1_ magnetic field was evaluated by acquiring an SNR map of a homogeneous H_2_O phantom using a 2 Tm^−1^ micro-gradient (Micro 5, Bruker), which was mounted on the Micro 5 probe base to accommodate the micro-NMR probe. The map was derived from two spin echo imaging experiments with a slice thickness of 0.1 mm and an in-plane resolution of (2 mm)^2^/(128)^2^ ≈ (15.6 μm)^2^, one containing the signal and the other one containing the noise information, according to [24]. The maps were calculated by taking the ratio of the signal scan and the mean noise amplitudes using MIPAV (National Institutes of Health, Bethesda, MD, USA). In the end, the data was exported and plotted using MATLAB R2014a (The MathWorks GmbH, Ismaning, Germany), shown in Fig 6a.

For a pre-defined homogeneous region with a maximum field deviation of ±5% with respect to the field in the center, resulting in a spherical volume with a diameter of 550 μm, we determined the SNR to be 24.91 ± 0.82 along the *x*-axis (Fig 6b) and 24.93 ± 0.98 along the *z*-axis (Fig 6c), while in both cases, the measured SNRs corresponds to the scaled, simulated *B*_1_-field profile of an ideal Helmholtz coil pair of same size.

The hermeticity and the bond strength of the ASSAIs were evaluated by performing a series of burst pressure measurements, were the chips were placed in a custom fixture with Luer connectors on both sides and sealed using NBR O-rings with 1 mm ID and 3 mm OD. One end was connected to the compressed air supply of the lab, while an end plug was placed on the Luer connector on the other side. The pressure was increased by cranking the handle until either the glass of the chip shattered or the bond delaminated, before recording the reading from the gauge. In total, 20 chips were measured, resulting in an average burst pressure and a standard deviation of (2.45 ± 0.25) bar.

### Homonuclear ^1^H spectrum

Shimming was performed on a H_2_O sample before performing the actual experiment. A single H_2_O resonance peak had a 50% linewidth of 0.62 Hz (35 Hz at 0.55% and 50.2 Hz at 0.11%). However, due to the wide-bore shim system employed, even large changes in shim currents had only a minor effect on the obtained linewidth.

**Fig 7:**
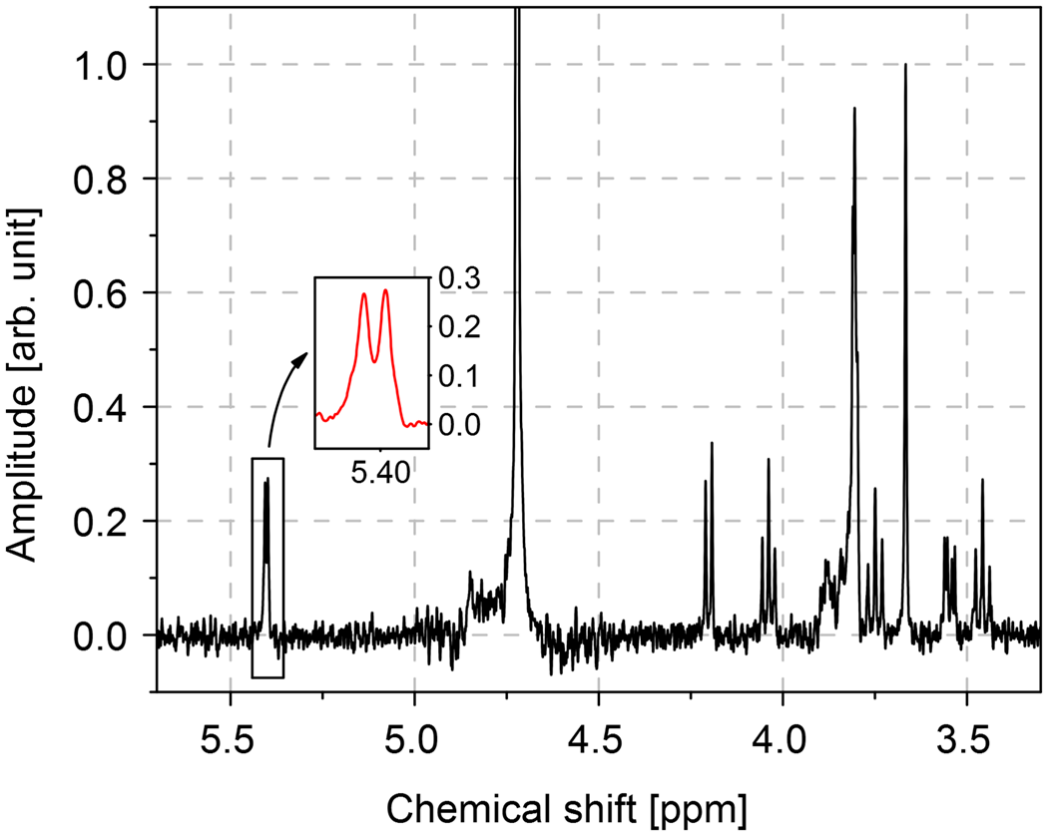
1D proton spectrum of 100mM sucrose in D_2_O. The inset shows the doublet of the anomeric proton.

A 1D spectrum was acquired from 100mM sucrose (molar mass = 342.3 gmol^−1^), dissolved in D_2_O. Using the observed volume *V*_obs_ ≈ 113 nL, this corresponds to a total amount of sample of 11.3 nmol, or a mass of 3.9 μg. The processed spectrum is shown in Fig 7.

The doublet of the anomeric proton, which appears at a chemical shift of 5.4 ppm [25] was successfully resolved, where we determined a coupling constant of 3.76 Hz (inset in Fig 7). The TSP peak at 0 ppm had a linewidth of 1.25 Hz (2.5ppb) before apodization and 1.75 Hz (3.5ppb) after.

### Heteronuclear ^1^H/^13^C experiments

**Fig 8:**
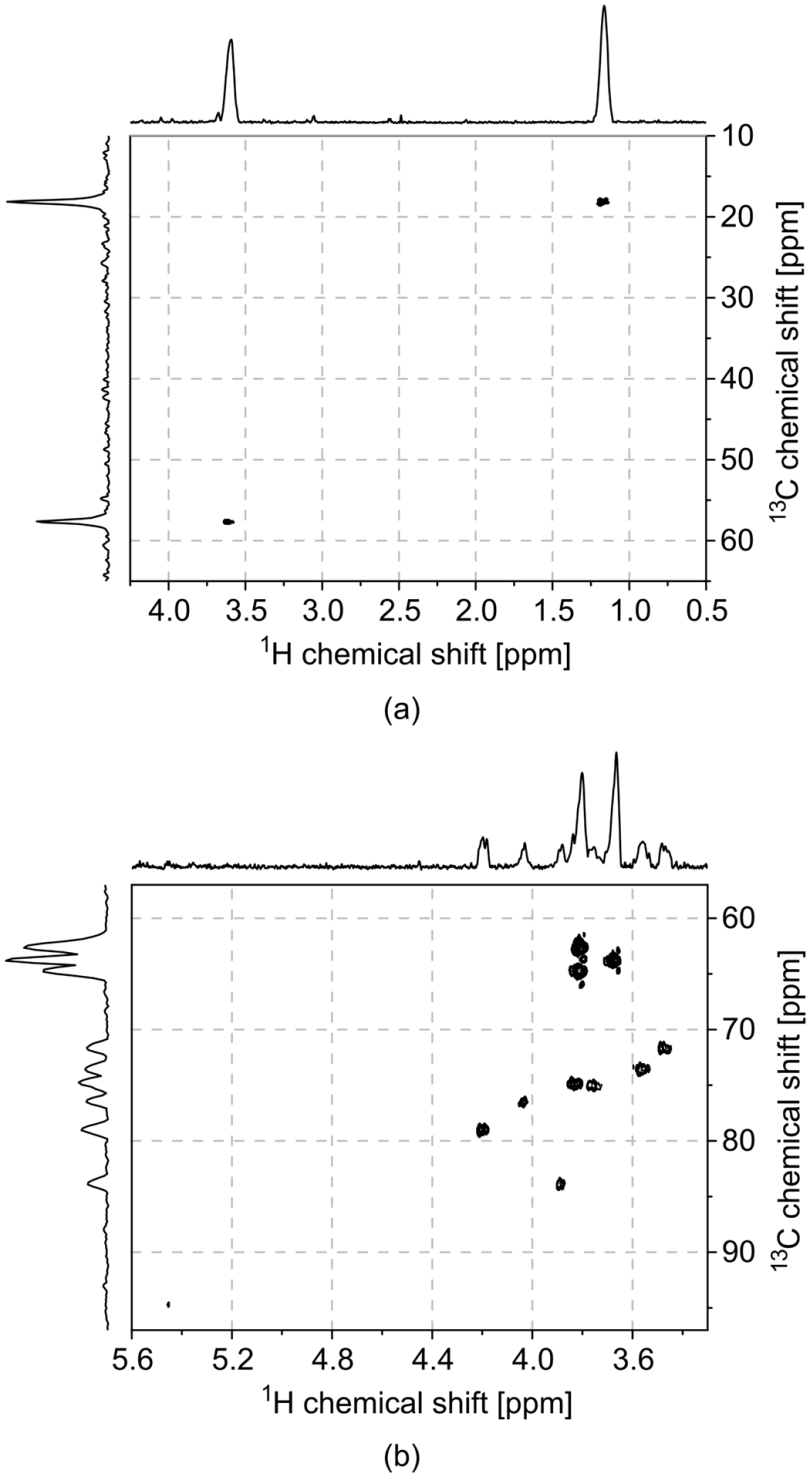
2D HSQC spectra. (a) Pure ethanol. (b) 1 M sucrose in D_2_O.

Two 2D HSQC spectra were acquired, the first of pure ethanol (CH_3_CH_2_OH) and in the second experiment, an ASSAI was primed with 1 M sucrose in D_2_O. The spectra are illustrated in Fig 8a and 8b.

All expected cross-peaks were successfully resolved in both experiments when compared to published spectra [25], demonstrating the ability of the detector to be used in structure elucidation, provided of course that the concentration of the sample is above the sensor’s limit of detection.

### ^1^H chemical shift imaging

Chemical shift imaging (CSI) combines localised spectroscopic information with an MR image, revealing spatially resolved chemical spectra. The method is mainly used for metabolic studies of plants or of model organisms in preclinical studies [26]. There is, however, high potential to expand the method towards technical applications, *e.g*., for the study of catalytic reactions in chemical reactions, or in battery research [27], and high spatial and spectral resolution may be achieved by employing sensitive micro RF probes.

**Fig 9:**
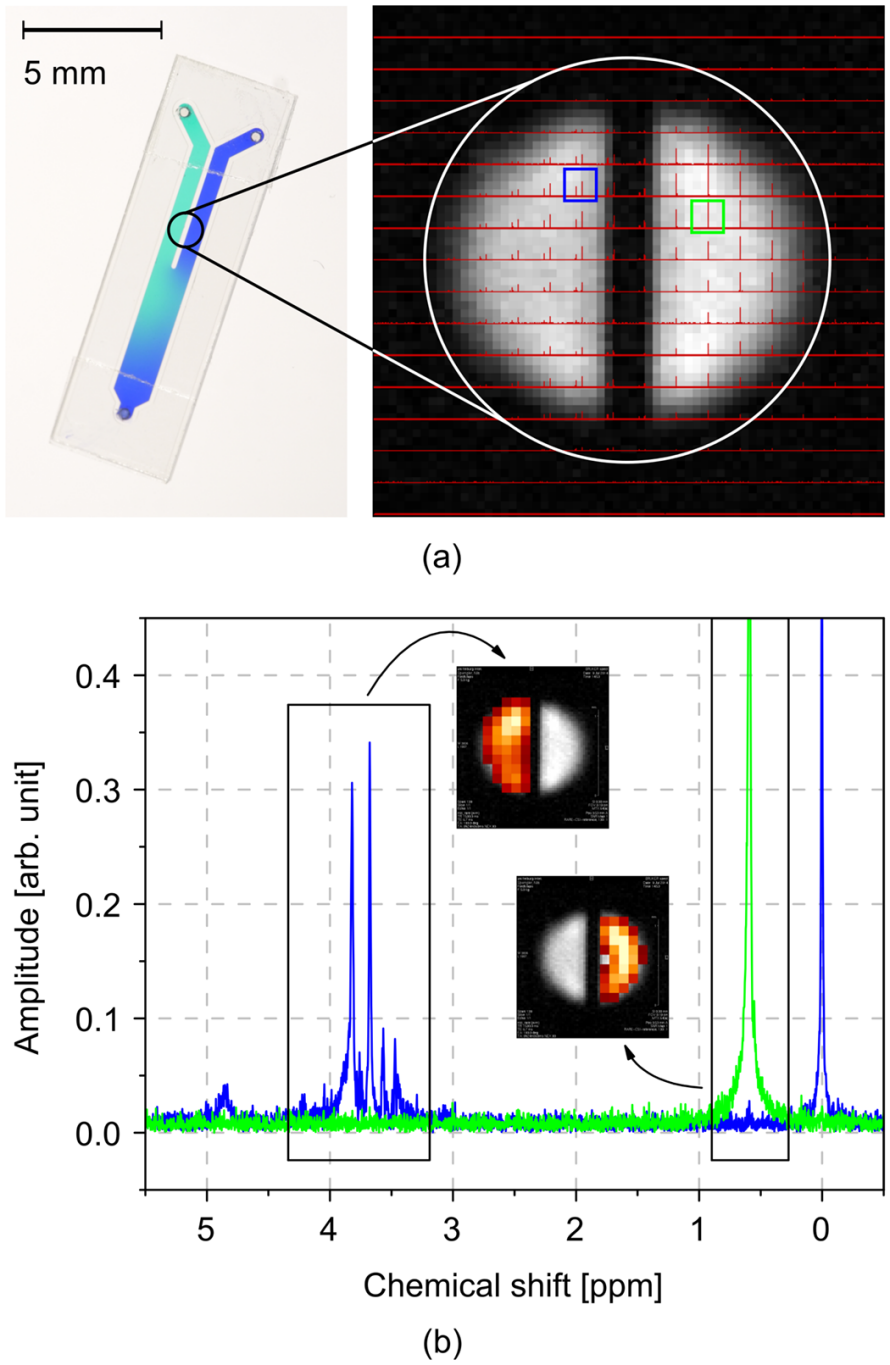
CSI-ASSAI, extracted spectra and assigned peaks. (a) CSI-ASSAI filled with aqueous ink (left) and overlay of morphological reference and CSI spectra (right). (b) Extracted and assigned spectra from the two voxels marked in the right half of (a).

For the CSI studies performed, a dedicated CSI-ASSAI was built, containing two microfluidic inlet ports, and one outlet port as shown in the left half of Fig 9a, where a CSI-ASSAI was primed with two different coloured aqueous inks. While the channels, and therefore the liquids, are separated in the first half of the chip, they merge and therefore mix in the second half.

A morphological reference image of a H_2_O phantom was first acquired in *t*_exp_ = 24 min using a RARE [28] sequence with a field of view (FOV) = 1.92 mm, a slice thickness of 0.1 mm, and an in-plane resolution of (30 × 30) μm^2^. The two channels were subsequently primed with a low viscous silicone oil (right channel) and a 1 M sucrose solution dissolved in deionized H_2_O (left channel), and TSP for referencing.

A CSI experiment was performed based on a point-resolved spectroscopy (PRESS) sequence, including water suppression (VAPOR) with an in-plane resolution of (120 × 120) μm^2^, which corresponds to a voxel volume of 1.44nL in *t*_exp_ = 1 h, 50 min. An overlay of the morphological reference and the CSI data is illustrated in the right half of Fig 9a. The spectra of the voxels marked by rectangular insets are illustrated in Fig 9b in more detail. Two groups of peaks have been marked in the spectra and were successfully assigned to the respective regions of the morphological reference using voxel-based integration of peaks before applying a heat colour look-up table.

As in this case both liquids are immiscible, the spectrum in each half of the chip corresponds to the one of the pure substances and therefore, peaks were assigned in either the left or the right chamber. Different intensities of the look-up table across the voxels can be attributed to (i) the grid of the matrix crossing the physical borders of the microfluidic channels and therefore voxels containing a reduced, effective sample volume, (ii) the inhomogeneity of the *B*_1_-field and therefore a varying sensitivity across the volume of interest and (iii) spreading or shifting of the spectrum, induced by deviations in *B*_0_-field which is caused by different susceptibilities in combination with insufficient shimming.

## Conclusion

The study reports on the redesign of a modular microcoil setup, composed of a wirebonded, micro Helmholtz coil pair and exchangeable, microfluidic sample containers (ASSAIs) to be used in magnetic resonance microscopy and spectroscopy. A complete redesign and simplification of geometrical features has lead to significant performance improvements over the first prototype [9], such as an 87% improved probe efficiency and an increased spectral resolution.

The round, microfluidic sample chamber was replaced by an elongated, rectangular-shaped chamber geometry that is oriented along the *B*_0_-field, similar to NMR tubes. The horizontally running microfluidic leads were omitted, while the microfluidic ports were moved further away to avoid material interfaces close to the sensitive volume of the microcoil. Doing so improved the H_2_O linewidth from 1.79 Hz (140.8 Hz at 0.55% and 208.9 Hz at 0.11%) to 0.62 Hz (35 Hz at 0.55% and 50.2 Hz at 0.11%) without the use of a susceptibility matching liquid. We successfully obtained a 1D homonuclear ^1^H spectrum of 100mM sucrose, which corresponded to a total amount of 11.3 nmol.

In contrast to our previous design, spectral peaks did not show distortions like peak-splitting, which can be attributed to fewer jumps in magnetic susceptibility near the site of detection. While in our case shimming had only little effect on the resolution of the spectrum due to the wide-bore shim system employed, efficient shimming may be achieved in the future by employing a micro shim system to encompass for the small volume of interest occupied by micro probes. The option to use passive shimming [29] remains open, so that the spectral resolution could be improved even further.

Besides, the improved sample containers can be sealed simply using Scotch tape due to the laser-drilled, microfluidic ports at the top, which allows storage of the sample for months without evaporation, while, for the former design, long-term sealing was difficult due to the side entrances and non-flush surfaces caused by dicing burrs. In contrast, the improved design enables gas-tight sealing using standard O-rings up to ∼ 2.5 bar and therefore straightforward external interfacing, which was not possible with the previous design. The yield of the wafer bond increased to 100% due to the reduced bond area and a more suitable resist.

Due to three measures, the fabrication yield of the micro-coil chip greatly increased and led to maximum deviations of the nominal dimensions < 2%, as (i) now the spacing of the coil pair is not defined by liquid photoresist but by the thickness of a dry film resist layer, and hence does not depend anymore on the exact amount of resist to be dispensed, or on levelling during curing. In addition (ii), the microcoils were suspended on a post base plateau to avoid the coils being pulled towards the substrate when the wedge bond is being formed. Finally (iii), the laser-cut polymer spacer between the coil pair was replaced by diced glass spacers, not being susceptible to melting during cutting, while mechanical alignment vastly simplified assembly of the layer stack.

We were able to demonstrate CSI of silicone oil and sucrose at voxel volumes of 1.44nL in < 2 H using a custom ASSAI. Spatial resolution may be improved at the expense of acquisition time and SNR, *i.e*., spectral resolution, and therefore remains a trade-off to be considered in prospective areas of application.

Integrating a trap circuit for the detector, thus avoiding the need for complicated decoupling that would be required for an arrangement with two separate coils, successfully enabled 2D ^1^H/^13^C heteronuclear HSQC NMR experiments of 1 M sucrose in D_2_O and pure ethanol, which, however, took several hours and is therefore limited to samples at high concentrations or in combination with labeled components.

Future improvements will include temperature stabilisation and will focus on the implementation of a third, lock-channel to track *B*_0_-field drifts, in order to avoid the smearing of spectral peaks, which would occur during long-term experiments as a consequence of the natural drift of the NMR magnet.

## Supporting Information

S1 Fig Trap circuit to realise a heteronuclear micro-NMR probe. The circuit provides a ^1^H and an X-channel.
(TIF)

S2 Fig SEM image of a microcoil, suspended on a post base plateau made from photoresist. The coil was wirebonded from 25 μm diameter insulated copper wire.
(TIF)

S3 Fig Polished cut of a Helmholtz coil pair chip. The slot in between the two coils was filled with epoxy before grinding and polishing. The measured distances (from left to right) were designed to be 600 μm, 550 μm, 600 μm, which results in deviations of 10 μm, 2 μm, 2 μm, *i.e*., errors of 1.7%, 0.4% and 0.3%.
(TIF)
