# Supplementary figures and images for: Heteronuclear Micro-Helmholtz Coil Facilitates µm-Range Spatial and Sub-Hz Spectral Resolution NMR of nL-Volume Samples on Customisable Microfluidic Chips
Source: PLoS One. 2016 Jan 5;11(1):e0146384. doi: 10.1371/journal.pone.0146384 (PMC4701473; doi:10.1371/journal.pone.0146384)

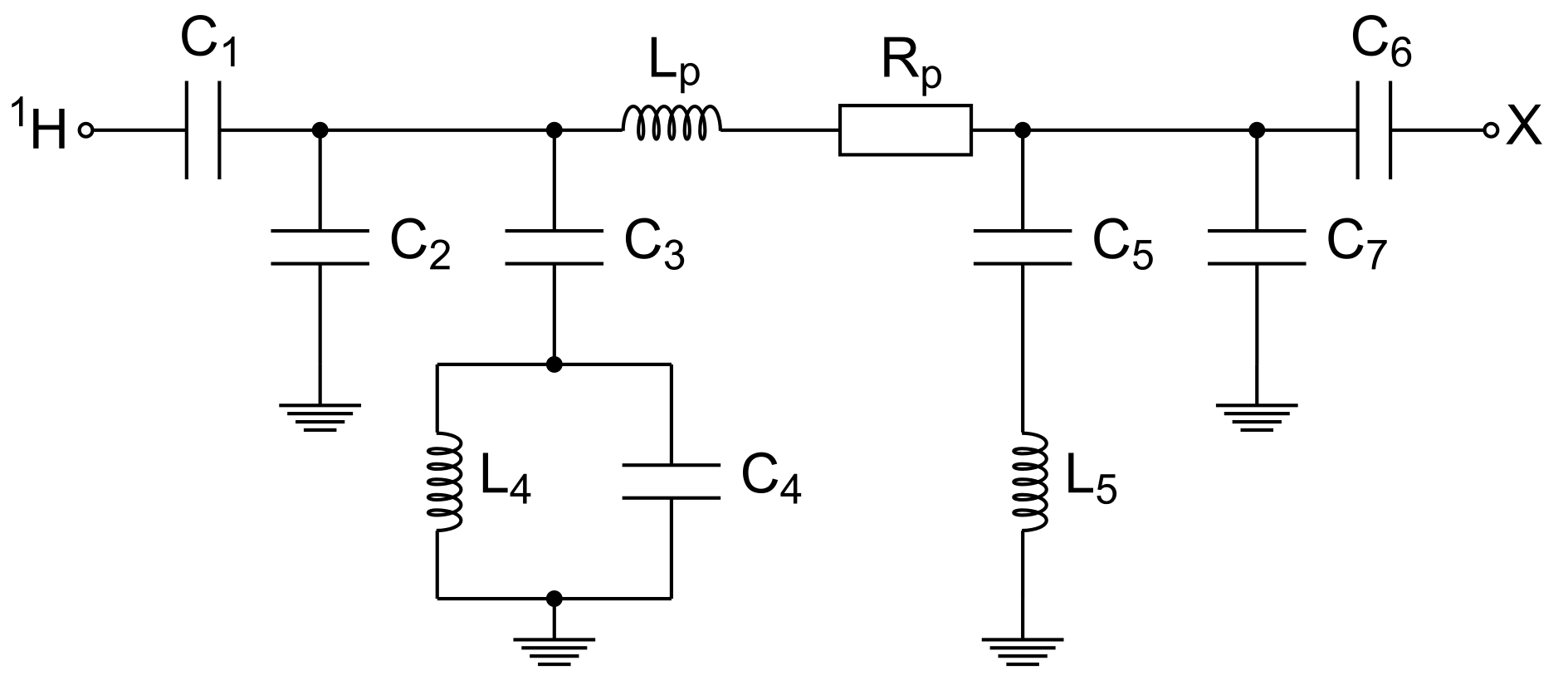

Supplement: S1 Fig — The circuit provides a 1H and an X-channel. (TIF) [file pone.0146384.s001.tif]

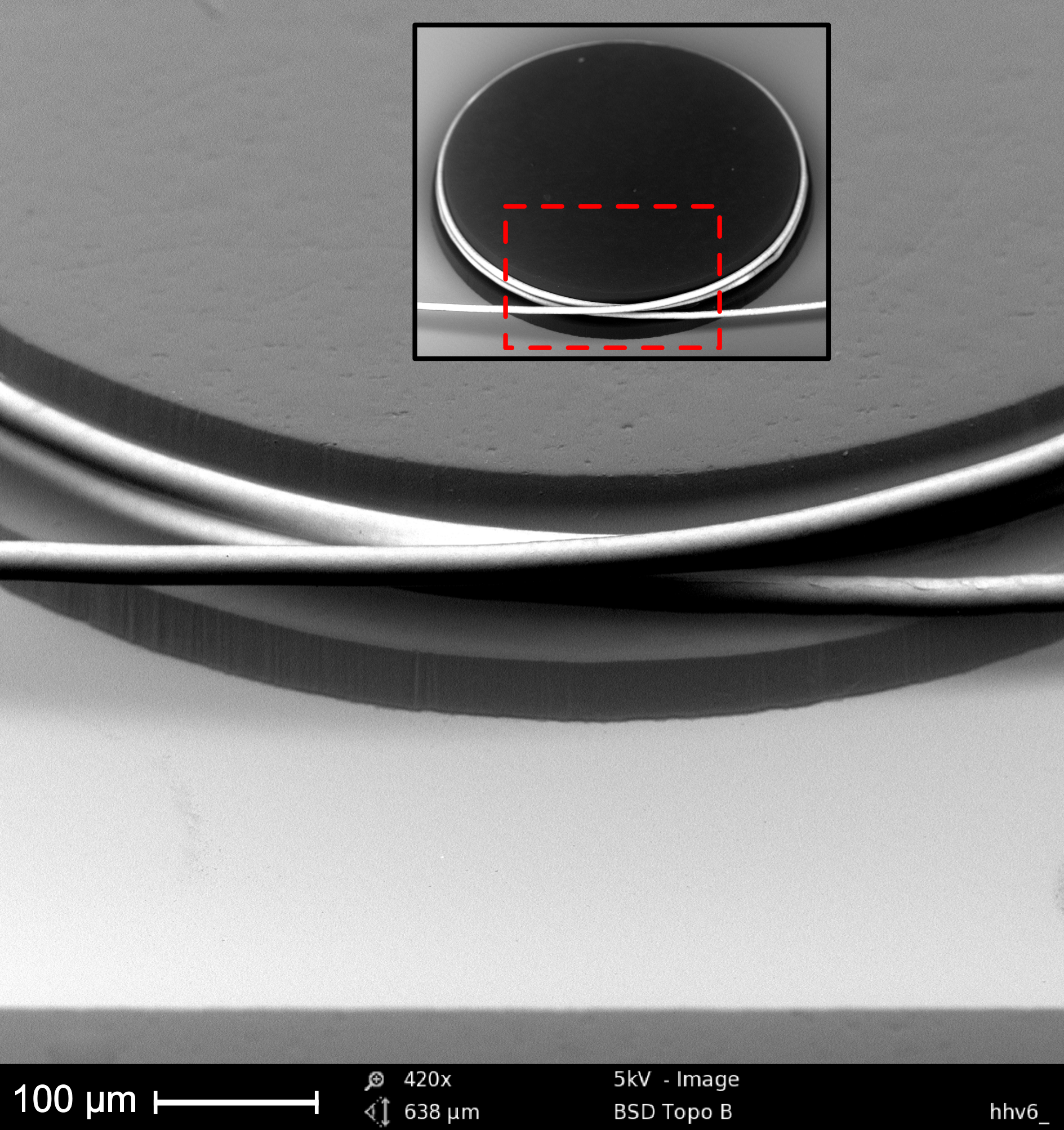

Supplement: S2 Fig — The coil was wirebonded from 25 μm diameter insulated copper wire. (TIF) [file pone.0146384.s002.tif]

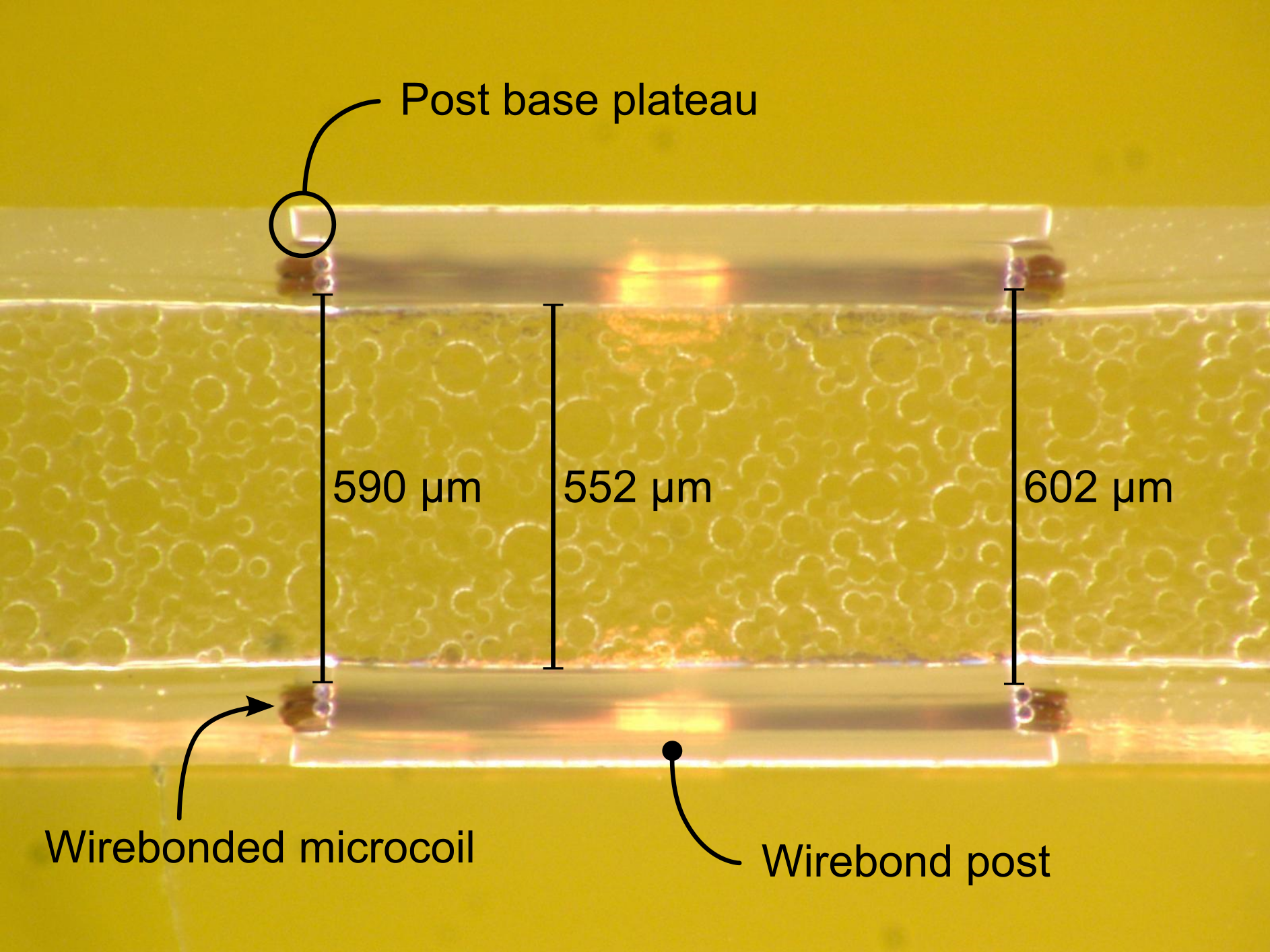

Supplement: S3 Fig — The slot in between the two coils was filled with epoxy before grinding and polishing. The measured distances (from left to right) were designed to be 600 μm, 550 μm, 600 μm, which results in deviations of 10 μm, 2 μm, 2 μm, i.e., errors of 1.7%, 0.4% and 0.3%. (TIF) [file pone.0146384.s003.tif]
